# Replication Pauses of the Wild-Type and Mutant Mitochondrial DNA Polymerase Gamma: A Simulation Study

**DOI:** 10.1371/journal.pcbi.1002287

**Published:** 2011-11-17

**Authors:** Zhuo Song, Yang Cao, David C. Samuels

**Affiliations:** 1 Center for Human Genetic Research, Vanderbilt University Medical Center, Nashville, Tennessee, United States of America; 2 Department of Computer Science, Virginia Tech, Blacksburg, Virginia, United States of America; UNC Charlotte, United States of America

## Abstract

The activity of polymerase γ is complicated, involving both correct and incorrect DNA polymerization events, exonuclease activity, and the disassociation of the polymerase:DNA complex. Pausing of pol-γ might increase the chance of deletion and depletion of mitochondrial DNA. We have developed a stochastic simulation of pol-γ that models its activities on the level of individual nucleotides for the replication of mtDNA. This method gives us insights into the pausing of two pol-γ variants: the A467T substitution that causes PEO and Alpers syndrome, and the exonuclease deficient pol-γ (exo^−^) in premature aging mouse models. To measure the pausing, we analyzed simulation results for the longest time for the polymerase to move forward one nucleotide along the DNA strand. Our model of the exo^−^ polymerase had extremely long pauses, with a 30 to 300-fold increase in the time required for the longest single forward step compared to the wild-type, while the naturally occurring A467T variant showed at most a doubling in the length of the pauses compared to the wild-type. We identified the cause of these differences in the polymerase pausing time to be the number of disassociations occurring in each forward step of the polymerase.

## Author Summary

As a polymerase copies a DNA strand, it may briefly pause. The pauses of the polymerase are important factors in the formation of large-scale deletions in mitochondrial DNA, a major cause of pathogenicity in mitochondrial diseases. To investigate these pauses, we have developed a simulation of the function of the mitochondrial DNA polymerase. As well as the wild-type polymerase, we consider two important mutant forms of the polymerase. Based on the simulation we predict that one of these two mutants would experience severe polymerase pauses, while the second would have only slightly longer pauses than the wild-type. This is important information for understanding the differences in pathogenicity between these two mutant polymerases. Furthermore, based on the simulation we determine that a particular reaction of the polymerase, the disassociation of the polymerase:DNA complex, is the most critical reaction determining the length of the polymerase pauses. Of all of the complex functions of the polymerase, the disassociation reaction has had the least attention experimentally and medically. Our simulation model points out that this neglected behavior of the polymerase should be examined more carefully in future research.

## Introduction

Mitochondria are essential for energy production through oxidative phosphorylation and have their own circular DNA (mtDNA) that is 16.5 kb in length. The proteins involved in mtDNA replication include the DNA polymerase pol γ and its accessory protein POLG2, the single stranded DNA binding protein (mtSSB), the mtDNA helicase (Twinkle), and a number of accessory proteins and transcription factors [1]. The minimal protein set required to replicate mtDNA in vitro include the two subunits of pol γ, the mitochondrial helicase and mtSSB [2]. However, in experiments measuring the kinetics of pol γ typically neither Twinkle nor mtSSB are added in the assay [3], [4]. Generally, these proteins are considered unnecessary since the kinetics assays only involve the replication of a short length of DNA.

The holoenzyme of human pol γ consists of a catalytic subunit (encoded by *POLG* at chromosomal locus 15q25) and a homodimer of its accessory subunit (encoded by *POLG2* at chromosomal locus 17q24.1) [5]. The catalytic subunit is a 140 kDa enzyme (p140) that has DNA polymerase, 3′-5′ exonuclease and 5′ dRP lyase activities. The accessory subunit is a 55 kDa protein (p55) required for tight DNA binding and processive DNA synthesis [6]. Two modes of mtDNA replication have been proposed; an asynchronous strand displacement model [7], [8] and a strand-coupled bidirectional replication model [9].

**Figure 1:**
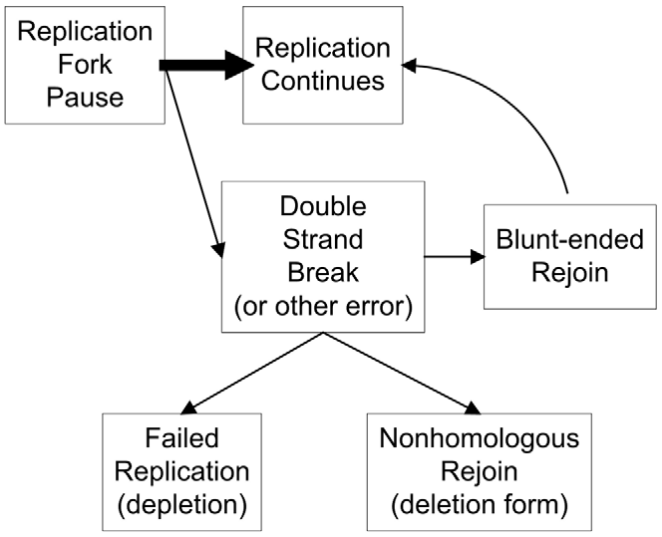
Conceptual diagram of possible consequences after mtDNA replication fork pauses.

The replication of DNA by a polymerase may not progress at a steady rate. How do replication pauses of pol γ relate to the commonly observed pathological phenotypes of mtDNA deletions and depletion? In theory, once a replication fork pauses, it can restart to continue the replication process without problems. However, if a pause is lengthy enough it might allow time for low-probability events such as double strand breaks. A double strand break can be repaired through blunt-ended rejoining, or homologous annealing of 5′- and 3′-repeat sequences [10], or nonhomologous end-joining [1] that will form deleted mtDNA. If the double strand break cannot be repaired, it will cause a failed replication. Repeated failed replications eventually would lead to depletion of the amount of mtDNA within the cell. These concepts are illustrated in Figure 1. Under this hypothetical mechanism, mtDNA deletions and depletion may both be possible outcomes from the same initial event, the pausing of the polymerase. Of course, other mechanisms for deletion formation are also possible [10].

Pathogenic mutations in *POLG* have been identified in patients with neurological or muscular diseases including progressive external ophthalmoplegia (PEO), Alpers syndrome, ataxia-neuropathy syndromes, idiopathic parkinsonism, and nucleoside reverse-transcriptase inhibitor (NRTI) toxicity [11], [12]. All these diseases were characterized by mtDNA deletions and/or depletion in symptomatic tissues [13]. PEO, as a mitochondrial disorder, is associated with the depletion of mtDNA and/or the accumulation of point mutations and deletions within mtDNA, caused by autosomal dominant mutations in *POLG* [14]. Nearly all of the adPEO mutations in *POLG* were located in the polymerase domain of pol γ. Alpers syndrome is a fatal early onset mitochondrial disorder characterized by tissue-specific mtDNA depletion [15]. In Alpers syndrome, the identified pol γ mutations are either homozygous A467T (MIM 174763.0002) or heterozygous A467T paired in *trans* with other mutations in *POLG* [16], [17].

The A467T mutation has been found in all of the major *POLG* related diseases, i.e. Alpers syndrome, ataxia-neuropathy syndromes and PEO [13]. The frequency of the A467T mutation varies from less than 0.2% to approximately 1% in different European control populations [18], [19], [20], [21]. In mitochondrial disease populations, however, the A467T allele was estimated to be the most common alleles associated with *POLG* diseases [13]. The homozygous A467T substitution of *POLG* has been found in several mitochondrial disorders with highly varied phenotypes. For example, in Alpers syndrome [22], [23], two unrelated teenage boys with homozygous A467T *POLG* had ataxia and severe epilepsy. In contrast, in a case of concurrent progressive sensory ataxia, dysarthria, and ophthalmoparesis [24], two siblings also homozygous for *POLG* A467T developed disease symptoms only late in life, with onset in their 40s. The kinetic parameters of the A467T mutant for both polymerase and exonuclease activity have been measured experimentally [15]. Compared to the wild-type catalytic subunit, the A467T substitution increased the *K_m_* of the enzyme 5-fold while also reducing the *k_pol_* value approximately 5-fold for DNA synthesis. As determined by the ratio of *k_pol_*/*K_m_*, the A467T substitution reduces DNA synthesis efficiency to 4% of the wild-type activity. In contrast, the exonuclease activity of the A467T variant is only decreased 2-fold compared to the wild-type [15]. Besides altering the kinetics of polymerase γ, the A467T variant in *POLG* also decreases the protein's physical association of the POLG2 subunit [15].

In addition to the identified pathogenic A467T substitution of pol γ in human, proof-reading deficient versions of the catalytic subunit of mtDNA polymerases have been created in two independent homozygous knock-in mouse models, one published in 2004 [25] and the other one in 2005 [26]. Both mouse models showed similar progeria-like phenotypes and shared the same D257A mutation on the second exonuclease domain of *PolgA* (the mouse homolog of *POLG*) that caused a profound reduction of the exonuclease activity but no decrease in DNA polymerase activity. Both mouse models [25], [26] were reported to have accumulated mtDNA point mutations. Using these two mouse models, other mitochondrial genome variations besides point mutation have been studied. For the first mouse model, after Trifunovic *et al.* [25] reported increased mtDNA deletion, Bailey *et al.* [27] using one- and two-dimensional agarose gel electrophoresis interpreted these non-replicating linear mtDNAs as the result of double-strand breaks of cyclic mtDNA caused by stalled replication intermediates. Ameur *et al.* [28] further stated that mutator mice have abundant linear deleted mtDNA molecules but extremely low levels of circular mtDNA molecules with large deletions. For the second mouse model, Williams *et al.* [29] applying next-generation sequencing to native mtDNA did not observe the accumulation of mtDNA deletions but instead reported multiple copies of the mtDNA control region in brain or heart. However, Vermulst *et al.* [30], [31] have identified mtDNA deletions as a critical driving factor behind the premature aging phenotype in these mouse models and have suggested there is a homology-directed DNA repair mechanism directly linked to mtDNA deletion formation. As this description shows, the data on the amount of deletions in these two mouse models is currently mixed and this is a research area that is rapidly developing.

**Figure 2:**
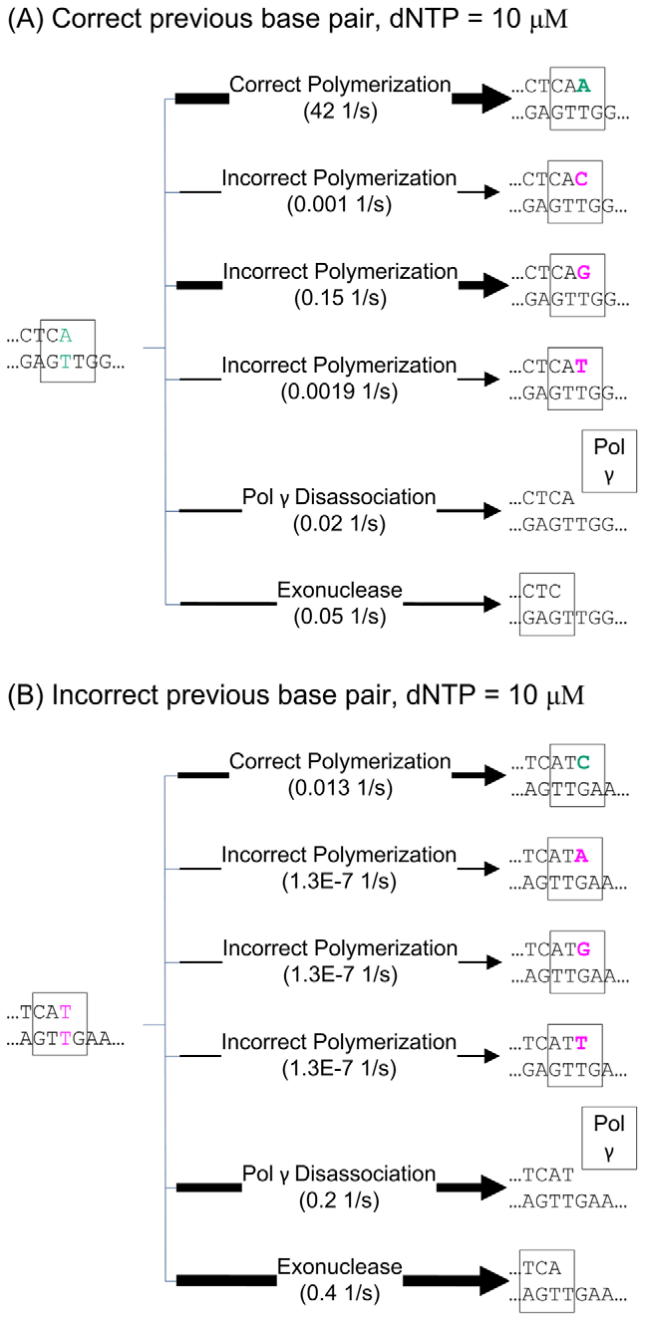
Diagrams of the six competing reactions of pol γ. Example reaction rates are given below each reaction name. The line size approximately represents the reaction rate. The bigger the reaction rate, the wider the line. (A) For a correct previous base pair, the highest reaction rate by far is the correct polymerization. (B) For an incorrect previous base pair, the highest reaction rate is the exonuclease reaction, allowing proofreading. All reaction rates in this diagram are calculated for dNTP pool levels equimolar at 10 µM.

To study the replication process of pol γ, we have built a computational model of the activity of pol γ [32], [33] (Figure 2), based on the experimental kinetic data. Our computational model of the mitochondrial DNA replication process was based on the Stochastic Simulation Algorithm [34], [35], a well-known Monte Carlo simulation method for chemical reactions. Using this stochastic simulation, we were able to quantify the pause lengths for the wild-type polymerase and for the pathogenic A467T and the exonuclease deficient pol γ variants.

## Results

We modeled three types of pol γ, the wild type and two mutants (the human pathogenic variation A467T and the completely exonuclease deficient form exo^−^). With frequencies of the A467T variant in different European control populations of 0.2–1% [18], [19], [20], [21], the naturally occurring heterozygous A467T alone, without paring in *trans* with other mutations in *POLG*, causes only mild, if any, pathogenic phenotypes [16], [17], [36], [37]. An exonuclease deficient POLG has never been reported naturally in humans or mice This severe POLG variant only occurs in the genetically engineered mouse models [25], [26]. Our hypothesis is that the pathogenic severity of a mutated pol γ depends on its susceptibility to replication fork pauses, not just its rate of point mutation formation.

### The single strand replication time

**Figure 3:**
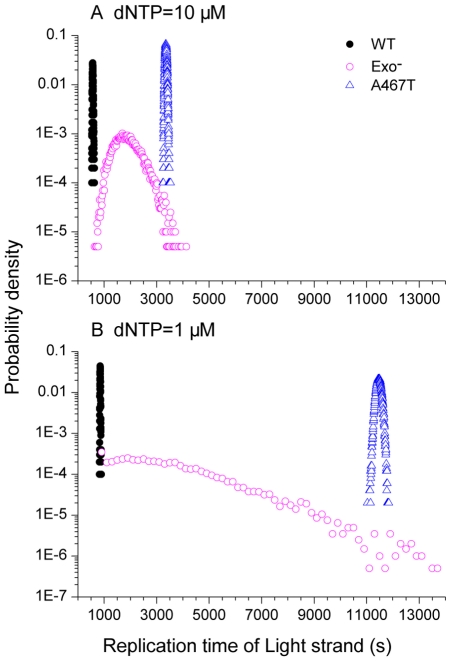
Probability distribution of the replication time of a single strand of mtDNA. In these examples, the light strand was used as the template. (A) dNTP pool levels equimolar at 10 µM, representing dividing cells. The median values of the replication time distributions of the wild type (WT, black dots), exo^−^ (magenta open circles), and the A467T variation (blue open triangles) are 563 s, 1837 s, and 3355 s respectively. (B) Equimolar 1 µM dNTP concentrations. The median values of the distributions of the WT, exo^−^, and A467T variant are 849 s, 2946 s, and 11465 s respectively.

We began by measuring the full replication time of a single strand of mtDNA in this computational model. The replication time of a single strand is defined simply as the time needed for the simulation model to complete the replication of all 16,571 base pairs in the mtDNA reference sequence. After running 10,000 simulations of replication of a single strand, the probability distributions of the replication time of wild-type and mutated pol γ are calculated (Figure 3). As expected from the enzyme kinetics, the naturally occurring mutant A467T simulation had a much longer replication time than the wild-type pol γ simulation. However, surprisingly, the severe pathogenic mutant exo^−^ had a highly variable replication time, ranging from times only slightly longer than that of the wild-type to replications taking longer than the A467T mutant simulation. In contrast, both the wild-type and A467T simulations had very tight distributions, meaning that the replication time had little random variation. Under the condition of dividing cells (Figure 3A, dNTP = 10 µM), the polymerase reaction rate of the A467T variant is approximately 7 times slower than the reaction rate of the wild-type pol γ. Considering the difference of kinetics values, the median strand replication time of 564 seconds for the wild-type and 3355 seconds for the A467T variant were reasonable.

Compared to dividing cells, postmitotic cells have lower dNTP levels and therefore pol γ in these cells has a lower polymerization rate and the replication process proceeds more slowly. Under this condition, we expected to see longer strand replication time (Figure 3B, dNTP = 1 µM). The median of strand replication time of wild-type pol γ increased 1.5-fold to 849 seconds, compared to the rate at dNTP = 10 µM. For the exo^−^ variant, the median replication time increased similarly from 1837 to 2946 seconds. For the A467T variant, the median replication time increased 3.4-fold to 11,465 seconds (3.2 hours).

### The time required for the slowest single forward step of the polymerase

The total replication time (Figure 3) is an average quantity, representing the summed effect of often tens of thousands of reactions. Pathogenic effects, such as deletion formation, may be more directly related to extreme single events that occur during the replication process than to this average quantity. To measure this, for each simulated mtDNA strand replication we recorded the time required for the longest single forward step of the polymerase. See the Methods section for a precise definition of this measure. We interpret these slowest forward steps in the polymerase activity as polymerase pauses.

**Figure 4:**
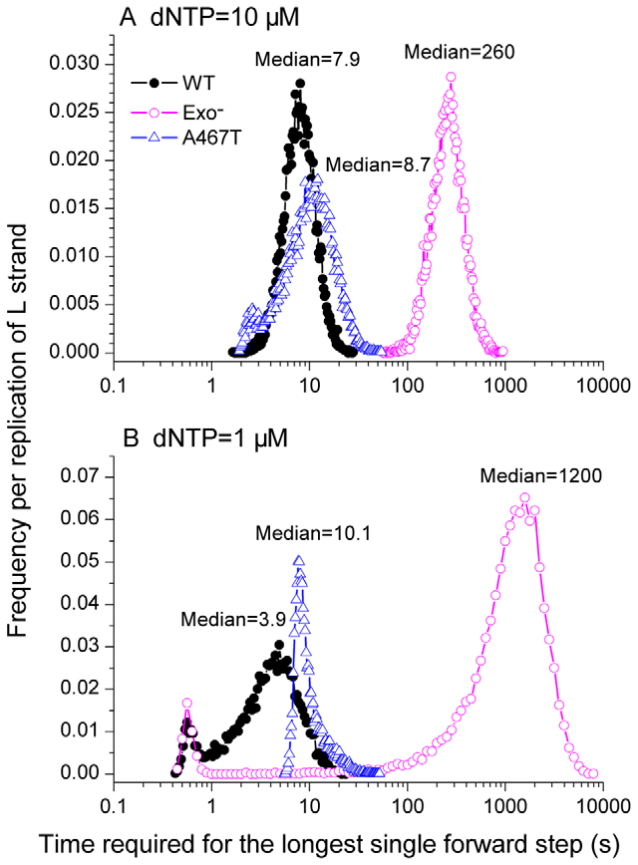
Frequency distribution of the time required for the longest single forward step. The median values of each curve are shown. (A) High dNTP level representing dividing cells. (B) Low dNTP levels representing postmitotic cells.

The exo^−^ simulation had extremely lengthy maximum single forward step times, corresponding to pol γ pauses (Figure 4). Compared to the wild-type, the exo^−^ polymerase had a 30 (Figure 4A) to 300-fold (Figure 4B) longer median of the longest single forward step time. However, the median of the A467T variant's longest single forward step time only increased slightly compared to the wild-type. This is reasonable because the slow polymerase reaction rate of A467T slowed down every forward step of the polymerase, without preferentially affecting the extremes.

For dividing cell conditions (Figure 4A, dNTP = 10 µM), the median time of pausing of the wild-type polymerase was 7.9 seconds, while it was a similar value of 8.7 seconds for the A467T variant. However, the median time of pausing of the exo^−^ polymerase was much longer at 260 seconds. For postmitotic cell conditions (Figure 4B, dNTP = 1 µM), the median time of pausing of the wild-type polymerase decreased to 3.9 seconds. The median time of pausing of the A467T variant increased slightly to 10.1 seconds, however, the exo^−^ pol γ had a dramatically increased median pausing time of 1200 seconds. Under both dNTP concentration conditions, the exo^−^ polymerase had the slowest “longest single forward step time”, implying the longest pauses and the greatest opportunity of deletions to occur.

### The reaction events in the longest single forward step

**Figure 5:**
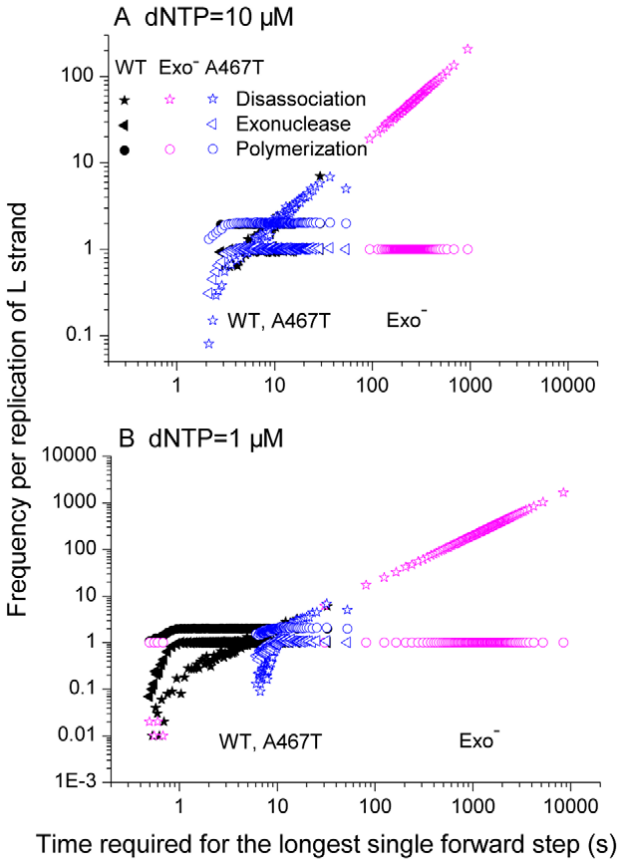
Events in the longest single forward step. For each pol γ variant, 10,000 simulations were carried out and the probability distributions of the events were calculated. Symbol colors represent the pol γ variants (black = wild-type, magenta = exonuclease deficient, blue = A467T). Symbols represent the reaction events (star = disassociation, triangle = exonuclease, circle = polymerization).

**Figure 6:**
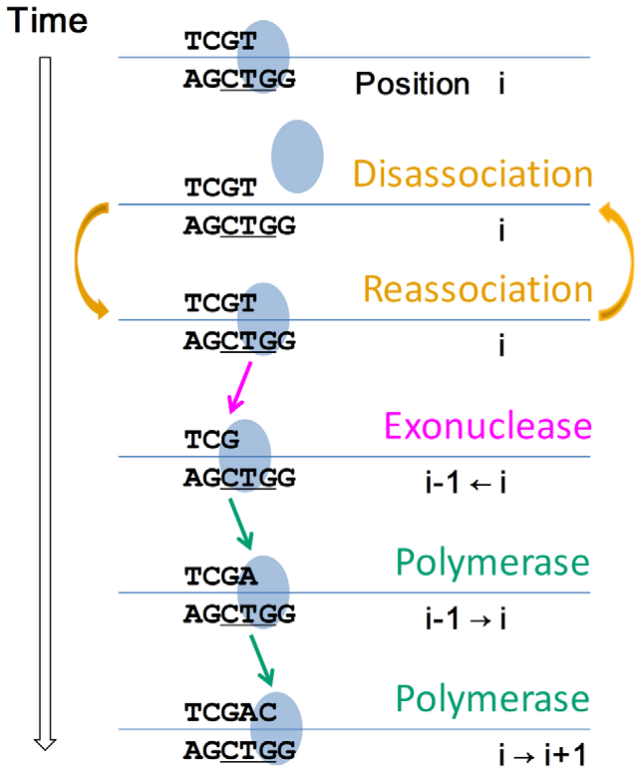
An example of events in a typical longest forward step of the polymerase. For the wild-type (WT) and the pathogenic A467T pol γ variant, a typical single forward step includes several dissociations and reassociations, one exonuclease reaction and two polymerizations.

We further identified what reaction events actually occurred in the longest single pausing step (Figure 5 and 6). The longest pausing steps usually occurred after incorrect polymerase events (Table 1), which inserted a non-Watson-Crick paired nucleotide into the new DNA strand. The exceptions to this (i.e., pauses after correct incorporations) were always very brief pauses, which can be seen as the small peaks to the extreme left side in Figure 4. In most of the cases of pausing after the incorrect nucleotide insertion, for both the wild-type and A467T variant, the longest single forward time step has one exonuclease reaction (to remove the incorrect nucleotide), two polymerase reaction (one to replace the removed nucleotide and a later one to move the polymerase forward to the next step), and a variable number of dissociation events (Figure 5). For the exo^−^ polymerase, there was one polymerase reaction and a variable but large number of dissociation events. In very rare cases, where pol γ replicated the whole strand without any error ever occurring, the longest single forward step time was very short and there was only one polymerase event and very few dissociation events.

**Table 1:** Strand replication time statistics.

Template	dNTP (µM)	Mean replication time (s)	Probability of incorrect previous base pair	Median longest single forward step time (s)
Light strand	1	849.4	92.69%	3.9
Heavy strand	1	813.2	95.80%	4.4
Light strand	10	563.8	100.00%	7.9
Heavy strand	10	542.7	100.00%	8.4

We found that the pausing time of pol γ was roughly proportional to the number of disassociations events occurring within that pause (Figure 5). The exo^−^ pol γ had extremely long polymerase pauses, with a 30 to 300-fold increase in the time required for the longest single forward step of the polymerase compared to the wild-type polymerase, while the naturally occurring A467T pol γ variant had only a slight increase in the length of the pauses compared to the wild-type pol γ. It is the number of polymerase disassociations occurring in each forward step of the polymerase that causes the differences. Due to the lack of competition of the exonuclease activity in the exo^−^ pol γ to remove the incorrect incorporation, disassociations of pol γ repeatedly recurred in each longest single forward step. The variation in the pausing time was almost completely due to variation in the number of disassociation events occurring during the pause (Figure 5).

### The effect of a partial loss of pol γ exonuclease activity

**Figure 7:**
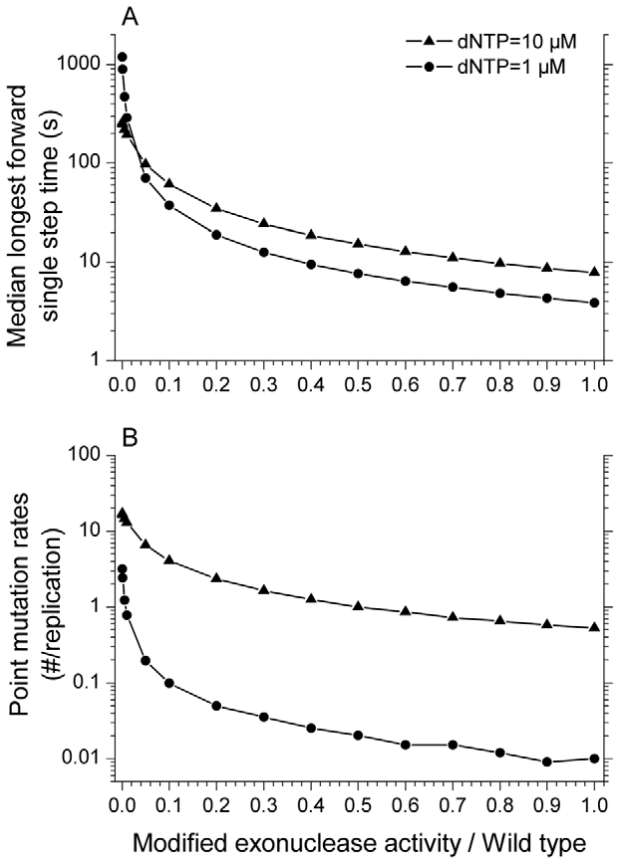
The effect of varied exonuclease activity. (A) Median longest forward time step occurring within a replication from the mtDNA light strand template (taken over 10,000 repeated simulations). (B) Point mutation rate per strand replication. With the decreasing of exonuclease activity from 100% activity to zero, both the median value of longest forward single step time and point mutation rate increased nonlinearly.

The exo^−^ mutant is an extreme alteration of the polymerase function, which we have modeled as the complete loss of exonuclease activity. What if a mutated pol γ lost exonuclease activity only partially, not completely? To answer this question, we decreased the exonuclease reaction rate in the simulation gradually from 100% activity, equivalent to the wild-type, to 0%, equivalent to the exo^−^ mutant simulation (Figure 7). We calculated the median longest single forward step time (Figure 7A) and the point mutation rate (Figure 7B) for both dividing and postmitotic cell conditions. These two quantities were chosen to represent the two main mechanisms for generating mtDNA damage from a defective polymerase: deletions driven by polymerase pausing and point mutations. Surprisingly, pol γ has to lose approximately 50 to 90% of its wild-type exonuclease activity before there are significant consequences shifting either of these measures by more than a factor of two.

## Discussion

The mitochondrial DNA replication time has been measured in dividing cells in cell culture, and approximately one hour is required for replication of both strands [38]. In our simulation, the light strand replication time under dividing cell condition (dNTP = 10 µM) was less than 10 minutes (Figure 3A), and the heavy strand replication required a similar time (Table 1). The standard asynchronous strand displacement replication model makes the double strand replication time (1+2/3) fold longer than that of single strand, where the 2/3 represents the fraction of the mtDNA sequence in the major arc between the heavy strand origin and light strand origin. Based on this estimate, the full mitochondrial genome replication time in our simulation was only 17 minutes. The simulated double strands replication time is 3–5 fold less than what has been reported in experiments [38]. This fast replication time is a direct consequence of the measured enzyme kinetics values for pol γ. However, the kinetic parameters we used were measured *in vitro* [3], [4], and the actual values *in vivo* may be different. Another reason why our simulated replication time is shorter than expected is that we could not include the reassociation time of pol γ with the DNA following a disassociation event, since there is no data on the reassociation rate. In addition, we cannot include the effect of the proteins mtSSB, Twinkle and other accessory proteins on the replication time, since these accessory proteins were not included in the basic experiments measuring the polymerase enzyme kinetics. All replication time in our simulation were underestimated for these reasons, which would make the pausing of pol γ in reality even worse than calculated from these simulations. Another relevant feature that cannot be included in this simulation is the role of DNA secondary structure in polymerase pausing. These secondary structures would cause additional pausing of the polymerase, beyond that calculated in this simulation.

The simulation results gave us a clear picture of how pol γ pauses may happen. For wild-type and the A467T pol γ variant, the longest single forward step always starts with an incorrect nucleotide incorporation. For example if this happens at position *i* along the DNA strand then an exonuclease event will happen with high probability at position *i*, removing the incorrect nucleotide, and the pol γ moves backward one step. After this, two following polymerase events are needed to move the polymerase forward to the position *i+1*. In this case, the longest single forward time step from position *i* to *i+1* requires at least three reaction events, one exonuclease reaction and two correct polymerizations, as well as any disassociation and reassociation events that may have happened between these events (Figure 6). In principle, there could be *N* exonuclease events and *N+1* polymerase events in each single forward step. However, 10,000 repeated simulations showed that there were almost always only one exonuclease event and two polymerase events (Figure 5). For the exo^−^ polymerase, once an incorrect incorporation happens at position *i*, the polymerase keeps repeating a disassociation-and-reassociation cycle until a polymerization happens and moves the polymerase forward to position *i+1* (leaving behind a point mutation). The disassociation-and-reassociation cycle repeats because the rate of polymerization following the incorrect nucleotide incorporation is very low. In the wild-type polymerase, the slow polymerization following an incorrect nucleotide incorporation allows time for the exonuclease reaction to occur with high probability, repairing the DNA replication error.

Several experiments [15], [39], [40] have measured the processivity of polymerase gamma. However, there is a fundamental difference between processivity as measured in these experiments and the concept of “polymerase pausing” as defined in our simulation. The polymerase processivity experiments [15], [39], [40] always use an unlabeled DNA trap to deliberately limit the polymerase to the very first disassociation event. At the first disassociation event the polymerase is captured by the DNA trap with very high probability and the polymerase reassociation is prevented, ending the DNA replication. In contrast, we define pausing as the longest time required to move the polymerase forward one nucleotide along the DNA strand, a process that we show in the simulation often includes several polymerase disassociation-reassociation cycles. Those extreme cases where multiple disassociation-reassociation cycles occur (by random chance) are the slowest step in the full mtDNA replication, which we take as a representation of polymerase pausing. As we show in Figure 5, the difference between the wild-type POLG and the exonuclease deficient POLG comes in the number of polymerase disassociation-reassociation cycles that occur in the slowest forward step of the polymerase on the DNA strand. This difference would not be seen in the processivity experiments where reassociation is deliberately prevented by the experimental design. Because of this fundamental difference, the processivity experiments are not in disagreement with this simulation model. A further difference between the processivity experiments and the polymerase pausing is that the processivity experiments only measure the behavior of the polymerase over hundreds to a few thousand bases [39], [40], while the polymerase pausing (as we define it) is a measure of the most extreme replication event (the slowest forward step) over 16,600 bases. Those rare events simply could not be observed in the much shorter processivity experiments.

A recent study [29] reported at least two orders of magnitude increase of point mutations in exo^−^ mice compared to controls in postmitotic tissues – brain and heart. In Figure 7B, under the postmitotic cell condition (dNTP = 1 µM), the point mutation rate with 100% wild-type exonuclease activity was 0.01 (number of uncorrected errors per strand replication) and the rate increased to 3.2 for the completely exonuclease deficient polymerase simulation. This is consistent with the experimentally measured difference between the exonuclease deficient mouse and the wild-type mouse [29]. In an exo^−^ mouse model [25], the measured mutation load of the exo^−^ polymerase γ was 3–13 mutations per 10 Kb (approximates to 5–21 mutations per replication) for brain and heart cells (which can be taken as postmitotic) and liver cells (which can be taken as mainly mitotic). This experimental data is consistent with our simulation results (Figure 7B), which shows 3.2 mutations per replication for postmitotic cells (dNTP = 1 µM) and 17 mutations per replication for mitotic cells (dNTP = 10 µM).

Based on this simulation model, a reduced DNA binding effect caused by variants in the accessory subunit might also be another reason to cause pausing of the polymerase. There is growing interest in variants in *POLG2*, the gene that encodes accessory subunit p55 of polymerase gamma [41]. Reduced DNA binding is not obviously related to mtDNA depletion, but our simulations clearly showed that the number of disassociation events that occur during the pause directly determines the length of the polymerase pause. Thus, based on this simulation model, reduced DNA binding of the polymerase to the mtDNA molecule could greatly increase the length of pauses of the polymerase both by increasing the probability of disassociation events and by slowing the rate of the reassociation reaction.

In addition to the altered mtDNA polymerization kinetics, the A467T variant in *POLG* also has altered kinetics in the formation of the protein complex with POLG2 [15]. We deliberately chose to model just the process of replication of the mtDNA, and not the preceding process of the formation of the polymerase protein complex from POLG and POLG2. Extending the model to include the stage of formation of the polymerase complex would greatly extend the complexity of the model, and would distract from the purpose of the model, which was to represent the process of mtDNA replication after replication is initiated. The kinetics values used in this model are from experiments [3], [4], [15], [39] using POLG and POLG2 together.

Several presumably pathogenic amino acid substitutions in pol γ have been identified in PEO patients, including G923D, R943H (neither are currently in OMIM) and A957S (MIM 174763.0014). Although all of these have been characterized biochemically [42], [43] with some limited kinetics, none of them has had its exonuclease activity measured yet. Without that critical data, we cannot simulate these variants. Recently, the pathogenic pol γ mutation Y955C (MIM 174763.0001), identified in Autosomal Dominant Progressive External Ophthalmoplegia, was reported to stall at dATP insertion sites [44]. Unfortunately, many important kinetic values of the Y955C pol γ variant are not available, such as the exonuclease and dissociation reaction rates, although its polymerization kinetics have been reported [42], [45].

## Methods

### Pol γ replication model

In this model, pol γ carries out four basic types of reactions: DNA polymerase activity, exonuclease activity, disassociation of the polymerase from the DNA, and reassociation of the polymerase with the DNA molecule (Figure 2). In the DNA polymerase reaction, pol γ adds one nucleotide to the new DNA strand. This nucleotide could be either a correct or incorrect (point mutation) base. In the exonuclease reaction, pol γ removes one nucleotide from the new DNA strand. The exonuclease reaction is an error correction mechanism, as the rate for removal of incorrectly incorporated nucleotides is much faster than that of correctly incorporated nucleotides, though the removal of both correct and incorrect nucleotides is allowed in the model. In the disassociation reaction, the polymerase separates from the DNA molecule. In the reassociation reaction (not shown in Figure 2), the polymerase re-attaches to the DNA molecule after disassociation.

At each position on the replicating mtDNA strand, pol γ randomly undergoes one of a series of six possible competing reactions: one correct polymerase reaction, three incorrect polymerase reactions, the exonuclease reaction, or disassociation (Figure 2). Which reaction pol γ undergoes at each time is determined by the propensity of each reaction, calculated using the reaction rates following the selection formulae as introduced in Gillespie [34], [35]. The simulation was implemented in C++ and run under LINUX.

Michaelis-Menten kinetics was used for all of the DNA polymerization reactions. The exonuclease reaction and the pol γ disassociation reaction were set to have constant reaction rates. For the two scenarios of a correctly inserted and incorrectly inserted previous nucleotide we had separate sets of kinetic parameters for each of the pol γ reactions taken from Lee and Johnson [46] and Johnson and Johnson [3], [4] (Tables S1, S2, S3, S4, S5). These studies have reported an increase in exonuclease and disassociation rates, but a decrease in incorporation rates by pol γ following an incorrect incorporation. This was included in the simulation model by using two sets of enzyme kinetics parameters, one set for reactions following a correct incorporation and another set for reactions following an incorrect incorporation. For an extended description of the simulation model, please see the Methods paper by Song and Samuels [32].

### Simulation method

In each simulation step, this algorithm calculates the propensity of each reaction and generates two random numbers to determine the time between reactions and the identity of the next reaction. The original Gillespie algorithm [34], [35] calculates propensities based purely on the substrate species' population (i.e. integer copy number). In our method, since we are only concerned with the dynamics of pol γ, we do not have to convert the state variable of every species to its copy number. In this way, the propensity functions are calculated from the same formula as the reaction rates for the involved competing reactions. These propensities were then used to randomly choose the sequence of reactions that occur, at the level of individual molecular reactions following the Gillespie algorithm.

### Pol γ kinetics parameters

Given the complexity of pol γ activity, it should not be surprising that even the large set of pol γ kinetic parameters that have been measured is still incomplete. The data used in Tables S1, S2 was measured only in the case where the previous base pair was an A:T. In principle the reaction rates could be different for other preceding bases, however in the absence of this data we have applied the measured reaction kinetics to all cases where the previous base pair is a standard Watson-Crick pairing. The data on kinetics following a non-Watson-Crick base pair are even more limited. The *k_pol_* and *K_m_* values reported [4] for this case were only determined for the correct pairing of a C opposite to a G in the template strand, where the previous incorrect base pairing was a T:T. We define an approximate model by setting the kinetic coefficients for all correct incorporations following an incorrect incorporation to be equal to this reported value (Tables S3, S4). Reaction kinetics for the incorporation of an incorrect nucleotide following an incorrect nucleotide are not available, to the best of our knowledge. Values for these parameters in the model (Table S3, off diagonal elements) were based on the observation from Table S1 that *k_pol_* values were approximately 1000 times less for non-Watson-Crick pairing compared to regular Watson-Crick pairings. Similarly, based on Table S2 *K_m_* values were estimated to be approximately 100 times greater for the non-Watson-Crick pairings and this was used to estimate *K_m_* values in Table S4 for the off-diagonal elements.

There are currently two main groups [4], [15] that have independently measured the kinetics of wild-type polymerase γ. One [4] employed pre-steady state measurement using single-turnover analysis to determine dNTP incorporation rate *k_pol_* and the dissociation constant *K_d_* of dNTP to bind to the polymerase-primer-template complex. The other one [15] employed steady state kinetic measurement that assumes that the complex of polymerase, primer and template behaves as a single enzyme and fit the data to Michaelis-Menten function to determine its *k_cat_* and *K_m_*. There are some disagreements of kinetic values between these two measurements and this inconsistency has recently been further addressed in the literature [45]. We chose not to mix values of wild-type polymerase γ from different measurement techniques, and instead we chose to continue to use the pre-steady state values [4], as we have in several previous publications.

However, the A467T variant kinetics have only been published using the steady state measurement technique [15]. To make the most reasonable estimation of the A467T kinetics based on published values consistent with the experimental methods [3], [4] used to determine the wild-type POLG kinetics, we followed the observation of Bertram *et al* [47] that even when polymerase enzyme kinetics measurements from different measurements were not in agreement, the dimensionless ratios of those kinetics constants often were in agreement. The A467T mutant enzyme is reported to have only 4% of wild-type DNA polymerase activity (the *k_cat_* decreased 5-fold and *K_m_* increased 5-fold compared to the wild-type) and 50% of the wild-type exonuclease activity [15]. We used these proportions to estimate the A467T variant kinetics based on the wild-type kinetics measured by pre-steady state methods [3], [4]. Finally, we modeled an idealized exonuclease deficient polymerase by setting the exonuclease activity to zero and keeping the other kinetics values at the wild-type values. The exonuclease deficient mice are reported to have normal DNA synthesis capacity, but no detectable exonuclease activity [25].

Data regarding the reassociation reaction rate is not available to the best of our knowledge. For this model we assume that once disassociation occurs the only possible reaction is then reassociation of the polymerase with the DNA template. Without reaction kinetics, the time required for this reaction cannot be calculated and is not included in this model. This approximation is not important to our results reported here on point mutation rates. The time required for reassociation would extend even further the pol γ pausing times that we estimate based on the computational model.

### Substrate concentrations

The polymerization reaction rates are functions of the deoxyribonucleotide triphosphate (dNTP) concentrations. However, concentrations of dNTP pools in mitochondria vary in different species, tissues, cell types and cell cycle phases. Measured dNTP pools in mitochondria of quiescent and cycling human fibroblasts are listed in Table S6 [48]. The units of picomoles per 10^6^ cells were converted to µM by using an assumed 200 femtoliters of mitochondrial volume per cell (0.2 femtoliters each mitochondria, 1000 mitochondria per cell) [49]. The four dNTP concentrations can be set individually in the model. In all cases reported here we have kept the four dNTP levels equal at concentrations of either 1 or 10 µM to approximately represent the conditions of postmitotic and dividing cells respectively [48]. The dNTP levels were held constant throughout the simulated mtDNA replication.

### mtDNA sequence

Vertebrate mitochondrial DNA has a highly asymmetric G content. The low-G strand is labeled the light strand, with the complement strand called the heavy strand. Sets of simulations were carried out separately for the light strand sequence (GenBank NC_001807) and the heavy strand sequence, formed by inverting and complimenting the light strand sequence. We found no significant difference in the results reported in this paper between the light strand and the heavy strand. The data reported here are based on replication of the heavy strand, which takes the light strand as the template.

### The time required for a single forward step

We define the time for pol γ to move from sequence position *i* to the next position *i+1*, which could include multiple intermediate reaction events, as the “single forward step time”. For each simulated mtDNA strand replication, there are 16,570 single forward steps and 16,570 time intervals. We choose the longest single forward step time to represent the worst polymerase pausing event that happened in the strand replication simulation.

### The number of simulations

We have three types of pol γ (the wild-type, A467T and exo^−^) and two cell type conditions (dividing and postmitotic). Since the model is stochastic, the results vary between runs with identical parameter values. For each one of the six scenarios, we ran the simulation 10,000 times to determine the probability distribution of the model results.

## Supporting Information

Table S1 Kinetic parameters *k_pol_* (1/s) for base pairings when the previously inserted nucleotide pair is a correct Watson-Crick pair. The identity of the template strand nucleotide is given in the rows (in the order A, C, G, T from top to bottom). The identity of the nucleotide inserted in the new DNA strand is given on the columns (in the order T, G, C, A from left to right).
(PDF)

Table S2 Kinetic parameters *K_m_* (µM) for base pairings when the previously inserted nucleotide is a correct Watson-Crick pair. The array order is the same as in Table S1.
(PDF)

Table S3 Estimated *k_pol_* (1/s) kinetic parameters for base pairings when previously inserted nucleotide forms a non-Watson-Crick pair. The array order is the same as in Table S1.
(PDF)

Table S4 Estimated *K_m_* (µM) kinetic parameters for base pairings when previously inserted nucleotide forms a non-Watson-Crick pair. The array order is the same as in Table S1.
(PDF)

Table S5 Reaction rates for the exonuclease and disassociation reactions of pol γ when previous base pair is a Watson-Crick pair or a non-Watson-Crick pair.
(PDF)

Table S6 Concentrations of dNTP pools (µM) in mitochondria of different human cells with citations.
(PDF)
