# Supplementary material for: Replication Pauses of the Wild-Type and Mutant Mitochondrial DNA Polymerase Gamma: A Simulation Study
Source: PLoS Comput Biol. 2011 Nov 17;7(11):e1002287. doi: 10.1371/journal.pcbi.1002287 (PMC3219627; doi:10.1371/journal.pcbi.1002287)
Supplement: Table S1 — Kinetic parameters kpol (1/s) for base pairings when the previously inserted nucleotide pair is a correct Watson-Crick pair. The identity of the template strand nucleotide is given in the rows (in the order A, C, G, T from top to bottom). The identity of the nucleotide inserted in the new DNA strand is given on the columns (in the order T, G, C, A from left to right). (PDF) [file pcbi.1002287.s001.pdf]

Table S1. Kinetic parameters k_{pol} (1/s) for base pairings when the previously inserted nucleotide pair is a correct Watson-Crick pair [1].

Base pairings	T	G	C	A
**A**	25	0.08	0.1	0.0036
**C**	0.012	37	0.003	0.1
**G**	0.16	0.066	43	0.042
**T**	0.013	1.16	0.038	45

The identity of the template strand nucleotide is given in the rows (in the order A, C, G, T from top to bottom). The identity of the nucleotide inserted in the new DNA strand is given on the columns (in the order T, G, C, A from left to right).

REFERENCES

1. Johnson AA, Johnson KA (2001) Fidelity of nucleotide incorporation by human mitochondrial DNA polymerase. Journal of Biological Chemistry 276: 38090-38096.
