# Supplementary material for: Replication Pauses of the Wild-Type and Mutant Mitochondrial DNA Polymerase Gamma: A Simulation Study
Source: PLoS Comput Biol. 2011 Nov 17;7(11):e1002287. doi: 10.1371/journal.pcbi.1002287 (PMC3219627; doi:10.1371/journal.pcbi.1002287)
Supplement: Table S2 — Kinetic parameters Km (µM) for base pairings when the previously inserted nucleotide is a correct Watson-Crick pair. The array order is the same as in Table S1. (PDF) [file pcbi.1002287.s002.pdf]

Table S2. Kinetic parameters K_m (μM) for base pairings when the previously inserted nucleotide is a correct Watson-Crick pair [1].

**Base pairings**	**T**	**G**	**C**	**A**
**A**	0.6	800	540	25
**C**	180	0.8	140	160
**G**	200	150	0.9	250
**T**	57	70	360	0.8

The array order is the same as in Table S1.

REFERENCES

1. Johnson AA, Johnson KA (2001) Fidelity of nucleotide incorporation by human mitochondrial DNA polymerase. Journal of Biological Chemistry 276: 38090-38096.
