# Supplementary material for: Replication Pauses of the Wild-Type and Mutant Mitochondrial DNA Polymerase Gamma: A Simulation Study
Source: PLoS Comput Biol. 2011 Nov 17;7(11):e1002287. doi: 10.1371/journal.pcbi.1002287 (PMC3219627; doi:10.1371/journal.pcbi.1002287)
Supplement: Table S3 — Estimated kpol (1/s) kinetic parameters for base pairings when previously inserted nucleotide forms a non-Watson-Crick pair. The array order is the same as in Table S1. (PDF) [file pcbi.1002287.s003.pdf]

Table S3. Estimated k_{pol} (1/s) kinetic parameters for base pairings when previously inserted nucleotide forms a non-Watson-Crick pair [1].

Base pairings	T	G	C	A
**A**	0.52	0.00052	0.00052	0.00052
**C**	0.00052	0.52	0.00052	0.00052
**G**	0.00052	0.00052	0.52	0.00052
**T**	0.00052	0.00052	0.00052	0.52

The array order is the same as in Table S1.

REFERENCES

1. Johnson AA, Johnson KA (2001) Fidelity of nucleotide incorporation by human mitochondrial DNA polymerase. Journal of Biological Chemistry 276: 38090-38096.
