# Supplementary material for: Replication Pauses of the Wild-Type and Mutant Mitochondrial DNA Polymerase Gamma: A Simulation Study
Source: PLoS Comput Biol. 2011 Nov 17;7(11):e1002287. doi: 10.1371/journal.pcbi.1002287 (PMC3219627; doi:10.1371/journal.pcbi.1002287)
Supplement: Table S5 — Reaction rates for the exonuclease and disassociation reactions of pol γ when previous base pair is a Watson-Crick pair or a non-Watson-Crick pair. (PDF) [file pcbi.1002287.s005.pdf]

Table S5. Reaction rates for the exonuclease and disassociation reactions of pol γ when previous base pair is a Watson-Crick pair or a non-Watson-Crick pair [1].

Previous base pairs	Exonuclease rate (1/s)	Disassociation rate (1/s)
Watson-Crick pair	0.05	0.02
Non-Watson-Crick pair	0.4	0.2

REFERENCES

1. Johnson AA, Johnson KA (2001) Exonuclease proofreading by human mitochondrial DNA polymerase. Journal of Biological Chemistry 276: 38097-38107.
