# Supplementary material for: Replication Pauses of the Wild-Type and Mutant Mitochondrial DNA Polymerase Gamma: A Simulation Study
Source: PLoS Comput Biol. 2011 Nov 17;7(11):e1002287. doi: 10.1371/journal.pcbi.1002287 (PMC3219627; doi:10.1371/journal.pcbi.1002287)
Supplement: Table S6 — Concentrations of dNTP pools (µM) in mitochondria of different human cells with citations. (PDF) [file pcbi.1002287.s006.pdf]

Table S6. Concentrations of dNTP pools in mitochondria of different human cells with citations [1].

dNTP (μM)	Quiescent skin fibroblasts	Cycling skin fibroblasts
dATP	5.6	14.3
dCTP	2.5	20.4
dGTP	1.3	4.6
dTTP	1.5	19.9

REFERENCES

1. Ferraro P, Pontarin G, Crocco L, Fabris S, Reichard P, et al. (2005) Mitochondrial deoxynucleotide pools in quiescent fibroblasts - A possible model for mitochondrial neurogastrointestinal encephalomyopathy (MNGIE). Journal of Biological Chemistry 280: 24472-24480.
